# Global Phosphorus Fertilizer Market and National Policies: A Case Study Revisiting the 2008 Price Peak

**DOI:** 10.3389/fnut.2017.00022

**Published:** 2017-06-14

**Authors:** Nikolay Khabarov, Michael Obersteiner

**Affiliations:** ^1^Ecosystems Services and Management Program, International Institute for Applied Systems Analysis (IIASA), Laxenburg, Austria

**Keywords:** phosphorus, fertilizer, market, global, policies, India

## Abstract

The commodity market super-cycle and food price crisis have been associated with rampant food insecurity and the Arab spring. A multitude of factors were identified as culprits for excessive volatility on the commodity markets. However, as it regards fertilizers, a clear attribution of market drivers explaining the emergence of extreme price events is still missing. In this paper, we provide a quantitative assessment of the price spike of the global phosphorus fertilizer market in 2008 focusing on diammonium phosphate (DAP). We find that fertilizer market policies in India, the largest global importer of phosphorus fertilizers and phosphate rock, turned out to be a major contributor to the global price spike. India doubled its import of P-fertilizer in 2008 at a time when prices doubled. The analysis of a wide set of factors pertinent to the 2008 price spike in phosphorus fertilizer market leads us to the discovery of a price spike magnification and triggering mechanisms. We find that the price spike was magnified on the one hand by protective trade measures of fertilizer suppliers leading to a 19% drop in global phosphate fertilizer export. On the other hand, the Indian fertilizer subsidy scheme led to farmers not adjusting their demand for fertilizer. The triggering mechanism appeared to be the Indian production outage of P-fertilizer resulting in the additional import demand for DAP in size of about 20% of annual global supply. The main conclusion is that these three factors have jointly caused the spike, underscoring the need for *ex ante* improvements in fertilizer market regulation on both national and international levels.

## Introduction

There were multiple economic impacts associated with the global financial crisis of 2007–2008. Market distortions were observable in oil and food prices, where oil was demonstrating a gradual increase over the 5 years preceding the crisis, whereas food prices, e.g., wheat price remained relatively stable and rocketed during the two crisis years showing a 100% increase (1). The scale of the rapid food price increase is vividly represented by the real food price index (RFPI^1^) calculated by the Food and Agriculture Organization of the United Nations (FAO). Over the two crisis years 2007–2008, RFPI increased at an average rate of 18% per year, which is the fastest annual growth observed over the past 55 years (1961–2015). The rapid growth in 2007–2008 is even more pronounced for dairy, cereal, and oil components of the RFPI (on average +27, +31, and +33% per year, respectively). An analysis of 2008 food crisis (2) points out the need to understand the connection between actual price changes and the impacts of fertilizer prices, behavioral responses to rising food prices, and government policies. In this context, however not directly linking to food prices, we analyze fertilizer markets, relevant policies, and also provide an approximate estimation of farmers’ response.

**Figure 1:**
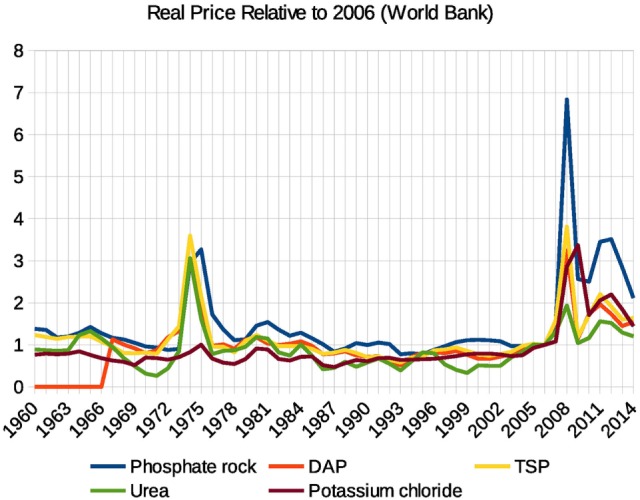
Historical price dynamics of phosphate rock, diammonium phosphate (DAP), triple superphosphate (TSP), urea, and potassium chloride (muriate of potash). Source: World Bank Commodity Price Data (The Pink Sheet), annual indices 1960 to present, real 2005 US dollars.

As modern agricultural producers rely heavily on fertilizer use to supply crops with necessary nutrients and achieve higher yields, the prices in food and fertilizer markets are naturally strongly correlated. In 2008, there was a price spike on the global fertilizer market as summarized by WBFPI—the World Bank’s fertilizer price index (3). The average annual WBFPI increase in 2007–2008 was +77% per year, which is well beyond the price jump observed in the food and oil markets, with a 2008 WBFPI spike of +120% from the 2007 price level. The 2008 real price increase as illustrated in Figure 1 was particularly strong in potassium and phosphorus fertilizers, when potassium chloride added +164% and phosphorus fertilizers diammonium phosphate (DAP) and triple superphosphate (TSP) added +108 and +140% to their 2007 levels, respectively (4). The most notable price spike far beyond those observed in food, oil, and ready-made fertilizer markets was in phosphate rock (PR), which is the main raw material for the production of phosphorus fertilizer: PR price added +352% to the 2007 level.

The global PR market is highly concentrated: according to 2008 and 2015 USGS reports, more than 90% of the total produced quantity is mined by a handful of countries (5, 6). The global reserves of scarce PR resources are also highly concentrated with about 75% of the world reserves concentrated in Morocco and Western Sahara, followed by 5% in China and 3% in Algeria (6). The biggest PR supplier on the world market according to the average annual data for 2005–2009 is Morocco with 11.7 Mtons/year followed by Jordan with 3.7 Mtons/year (1). Here and further, we abbreviate million tons with megatons. The biggest PR consumer on the world market according to the average annual data for 2005–2009 is by far India with 4.9 Mtons/year followed by USA with 1.7 Mtons/year (1). In the ready-made fertilizer trade (including DAP, TSP, and monoammonium phosphate—MAP), the share of DAP exceeds 50% when accounting for P as a nutrient (7). In the years preceding and following 2008, the biggest seller and buyer of DAP on the international market were, respectively, the USA and India both with about 5 Mtons of DAP traded annually (1).

In the context of the 2008 phosphorus fertilizer price spike, the aim of this research is to identify major factors that triggered the price peak and those that pushed the price higher up. The essential motivating questions are why India doubled imports of P-fertilizer (DAP) in 2008 when the price had doubled and if this increase of DAP importation by India could have caused or substantially contributed to the price spike.

## Materials and Methods

The following analysis, framed within the agricultural development trends in India preceding 2008, provides a review of potential global market price drivers with added quantitative analytics and concludes with the exploration of Indian policies in domestic agriculture and fertilizer industry.

### Trends in Indian Agriculture

**Figure 2:**
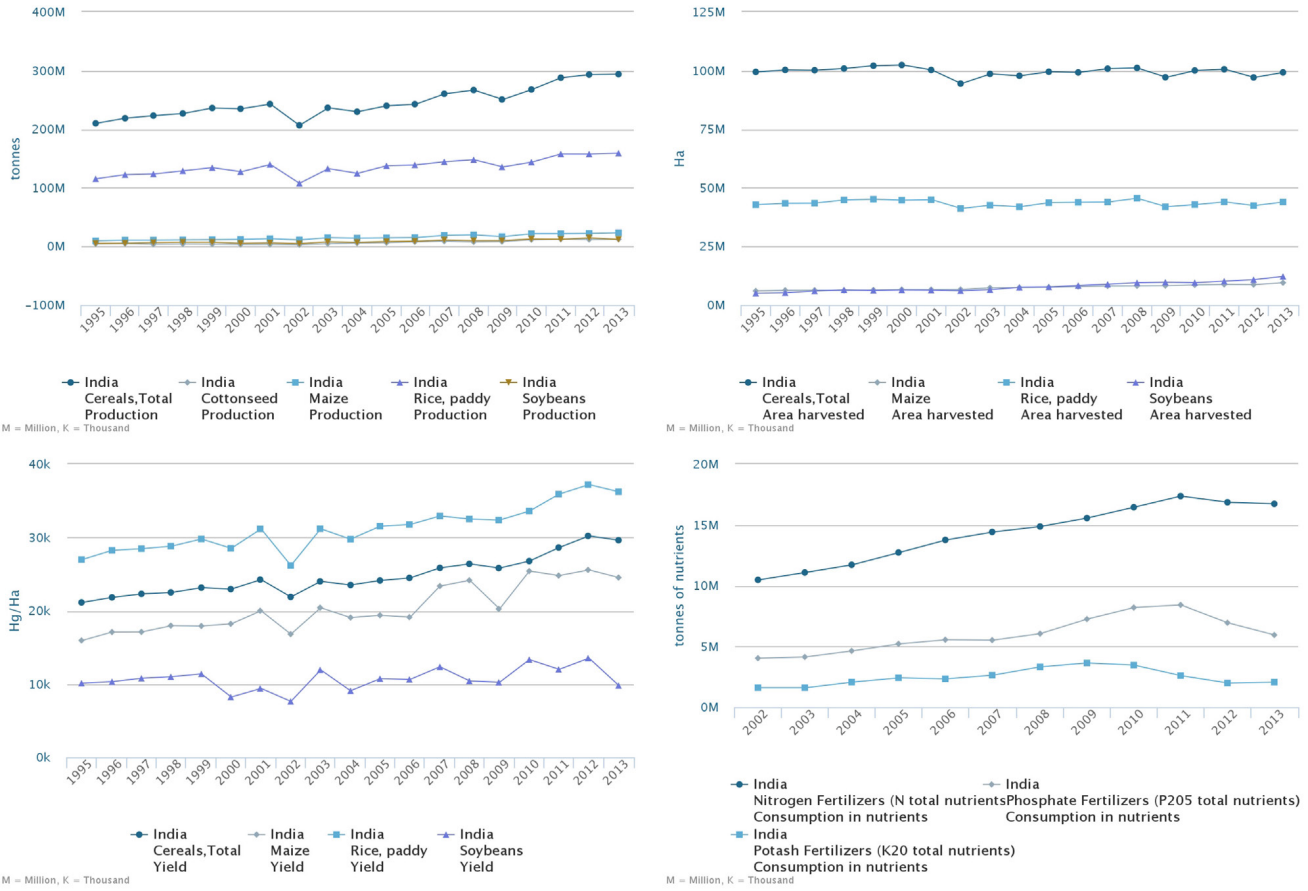
India’s cereals production (top left panel), harvested areas (top right panel), crop yields (bottom left panel), and fertilizer use (bottom right panel). Source: FAOSTAT.

India’s agricultural production is demonstrating a constant increasing trend since mid-1990s (8). According to FAO data, the harvested area for cereals remains nearly constant with the exception of soybeans and maize that is still marginal in relative terms (8). As illustrated in Figure 2, crop yields grew, supported by substantial intensification through a higher usage of fertilizer (8). The increase in efficiency of agricultural production allowed India to reduce import of cereals and become one of the major world exporters: the net export of cereals increased from almost 0 in the late 1990s to 10–20 Mtons in 2011–2012 (8).

According to FAOSTAT (8), the total value of fertilizers imported by India increased from 0.6 bln US$ in 2002 to 4.9 bln US$ in 2007 and 13.3 bln in 2008; the net export value of agricultural goods increased from 1.5 bln US$ in 2002 to 8.6 bln US$ in 2007 and 8.2 bln US$ in 2008; and domestic food consumption (calories per capita) increased in the period 2002–2008. Until 2007, intensification through greater fertilizer use was paying off and from this perspective the Indian strategic commitment to agricultural intensification for meeting domestic demand and increasing export revenues seemed to be well founded. However, in 2008, the cost of fertilizer imports exceeded the agricultural export value.

### Global Fertilizer Price Drivers

As there is a considerable economic impact of the 2008 fertilizer price peak on the economics of Indian agricultural sector, this section provides a reflection on the global market price drivers relevant to the observed price peak and widely quoted in the literature (9–13). These drivers are assumed to have jointly produced the observed effect; however, as we highlight below, the relative contribution of individual drivers is likely to be very different.

*The falling trend of the value of the US dollar* was observed over the period 2003–2008, when the US dollar depreciated against the Brazilian real (48%), Canadian dollar (34%), the Russian ruble (23%), the Indian rupee (18%), and the Chinese yuan (13%). This is assumed to have led to a rising demand in some of these countries as the imports became cheaper. According to our analysis, the influence of this factor on the explored fertilizer price is rather moderate for a number of reasons. First, the exchange rate influence is not sector specific at a global scale. Second, fertilizer imports for agricultural products to be consumed domestically could have indeed become cheaper, whereas this is less true regarding exports traded against the US$. Third, the moderate shift in exchange rate is unlikely to have produced a price spike in fertilizers of the observed magnitude.

*A spike in transportation costs* due to the increase in fuel prices and high demand for freight services during 2007–2008 is considered as another contributor to the 2008 fertilizer price spike. According to our assessment, similar to the exchange rate, this influence is rather moderate. Using US RMOC Freight Rate Index as a proxy (14), one could conclude that the freight cost at peak in 2008 had a relatively small increase of 25% compared to 2007. Another valuation of the transport cost increase and potential impact this caused could be made employing the data from the UN Comtrade database (1). The analysis of the global annual traded quantity in PR and the respective reported trade values of the export and import leads to an estimation of average transportation costs of about 25 US$/ton of PR in 2005–2007, whereas in 2008, the estimated transportation cost indeed doubled and exceeded 50 US$/ton. However, the average PR import price increased, respectively, from 70 to 80 US$/ton to almost 220 US$/ton in 2008, meaning that the share of the transport cost in the import price *dropped* from almost 40% to below 25%. This consideration makes it clear that even though transport costs have contributed to the price increase, this was rather a marginal factor.

**Figure 3:**
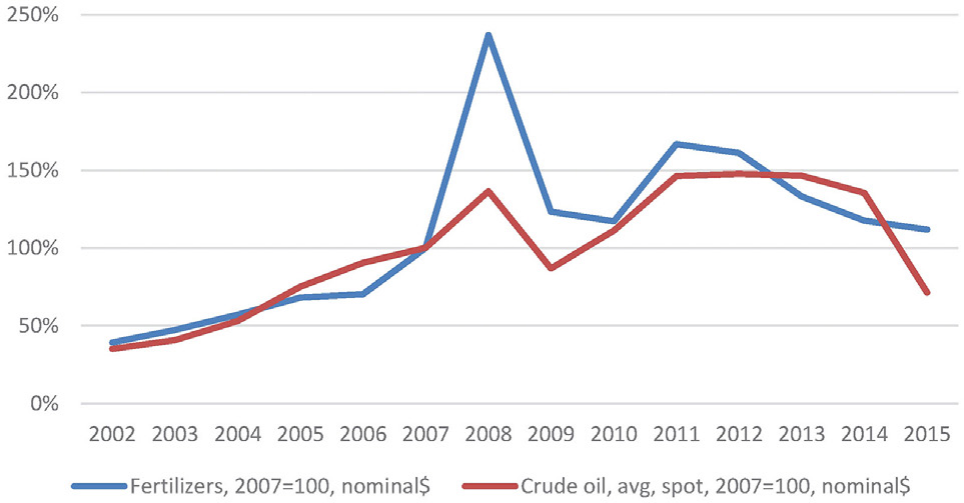
Global fertilizer and oil prices in percent relative to 2007. Data from database: Global Economic Monitor (GEM) Commodities, World Bank.

*An increase in oil prices* is deemed to have contributed to the fertilizer price spike. According to our analysis, in relation to phosphorus fertilizer and the 2008 price peak, this was rather marginal contribution. The observed response of fertilizer prices to oil prices (3) is rather disproportionate: the relative increase in oil prices in 2008 compared to 2007 was about +35%, whereas the increase in fertilizer prices over the same period was about +135% as illustrated in Figure 3.

*An increase in fertilizer demand for biofuels production* in the US, Brazil, and Europe is often mentioned among the factors contributing to the price spike in 2008. Even though from 2007 to 2008 there was a large +33% increase (+10 mln ha) in the area under energy crops (15), the total 2008 global production increase in biofuels was only about +5%, adding 50–1,150 Mtons in oil equivalent (15). On global scale, there was no visible expansion of the agricultural area that remained constant at about 5,000 million ha over the entire 2000–2008 period according to FAOSTAT (8). Hence, one cannot expect an extreme change of fertilizer consumption in 2008 explained by biofuels.

*Agricultural commodity prices* almost doubled in the 2 years 2007 and 2008, leading to increasing profits of farmers and hence are assumed to have stimulated higher demand. We find that food price increase alone seems to be a rather moderate factor in terms of scale of the impact on fertilizer price. This argument is supported by two facts. First, farmers in many food exporting countries enjoyed high price levels for their production, yet only India experienced a dramatic increase in the quantity of imported fertilizer. Second, the food price peak is less pronounced than that of fertilizer, adding about 18% in price annually in 2007–2008 as described by FAO’s RFPI.

*Low inventories* at the beginning of 2008 and the limited capacity of the fertilizer industry to rapidly adjust to a surging demand are claimed to have greatly influenced the market. As it regards inventories, there is unfortunately no information on global scale and an attempt to produce an estimate based on the FAOSTAT data (8) for India for 2002–2013 by taking into account production, consumption, and trade quantities expressed in P_2_O_5_ total nutrients and assuming 0 balance in 2002 uncovered an inconsistency in data leading to an accumulated balance deficit of 6 Mtons of P_2_O_5_ equivalent over the time period of 2002–2013. This P-imbalance is equal to the average annual consumption of India over the time period of 2002–2013. The fact that the fertilizer industry did not cope with increased demand is either an indicator of bad planning of supply, some unforeseen extreme changes in demand or/and desire to increase profits by exercising the market power. All these aspects may have played a role, as we discuss below.

*Increasing raw material costs* in 2007–2008, e.g., PR prices almost tripled are named among the other factors contributing to fertilizer price increase. Regarding specifically the phosphorus fertilizers, the principal raw material PR is almost fully consumed by the fertilizer industry and ultimately lands on the fertilizer market, this is why the extreme PR price increase cannot be considered as an external factor to the industry where PR mining is effectively part of it.

Even though an *increasing concentration of market power* in a few major countries and companies is certain, this fact alone is not likely to have triggered the 2008 price peak. However, it could have magnified the peak. China, as one of the largest phosphate exporters in the world in the years 2005–2006 preceding the crisis, supplied about 5% of PR and about 7% of DAP to the global international market and in 2007 China even increased its DAP share to 19% while decreasing its PR share down to 3% according to the UN Comtrade database (1). In April 2008, China introduced an export tax of 100% on fertilizers to ensure that domestic production was used within China (10). This led to a correction of the Chinese share in DAP on international market down to 8% (still higher than in 2006), yet slightly increased its PR share from 3 to 7% as reported in Comtrade (1). Overall, out of the 19% drop in global phosphate fertilizer export in 2008 (P_2_O_5_ equivalent), only 7% can be attributed to China according to a report based on the data from International Fertilizer Industry Association (IFA) (16). This is a clear indication that it is not only China who had decreased exports in favor of protecting domestic consumption. At this point of the analysis, the protective reaction of fertilizer supplying countries seems to be a plausible price spike magnifying mechanism: in anticipation of a high export price, national authorities protect domestic consumption by reducing the export, hence tightening the supply, and in so doing, increase international prices even more.

Another factor mentioned in the literature is that *major international buyers subsidize domestic use of fertilizers, and this is the reason for farmers being slow in reducing consumption* in response to a price increase. This component of the price spike magnifying mechanism is discussed below together with its plausible trigger.

### Fertilizer Policies in India and Their Implications

As we discussed above, over the past years, India has increased agricultural production through intensification, where fertilizer application plays an important role. Agricultural production is important for meeting the demand of domestic consumption and is a substantial part of Indian export. The total share of agriculture in Indian GDP is about 18% according to the World Bank data (17). This share is relatively large compared to other countries: in China, the respective share is below 10%, in Russia below 6%, and below 2% in the USA and Austria (17). To support agriculture as a major sector in Indian economy, the government provides domestic farmers with fertilizer subsidies.

There have been several fertilizer subsidy schemes implemented in India over past decades, and they vary both by the fertilizer type and price setting mechanism. An exhaustive authoritative overview is provided by the Department of Fertilizers of the Ministry of Chemicals and Fertilizers of India (18). Here, we highlight some of the information relevant specifically to P-fertilizers. First, according to the Comtrade data (1), PR and DAP are the major sources of P in Indian import, whereas MAP accounts for about 10% of volume and value of DAP. The DAP-related policy in force in 2002–2010 was essentially a price cap provided to a farmer by the government in the form of guaranteed maximum retail prices (MRPs). According to the subsidy scheme, “the difference in the delivered price of fertilizers at the farm gate and the MRP was compensated by the Government as subsidy to the manufacturers/importers” (18). This scheme is effectively fixing the price for domestic farmers and isolating them from any negative impact of increasing prices on international fertilizer markets. Moreover, if the food prices start to increase, the farmers have an incentive for buying extra amount of fertilizer in order to obtain higher yields (or expand area) and export products in larger quantities, maximizing their revenues at a minor cost. A moderate increase in food prices could be a sufficient incentive to increase fertilizer use under these conditions, leading to a demand increase where the fertilizer purchasing price is virtually unlimited. This forms the consumer side of the fertilizer spike magnifying mechanism, exacerbating effects of fertilizer export restrictions on the producer’s side.

**Figure 4:**
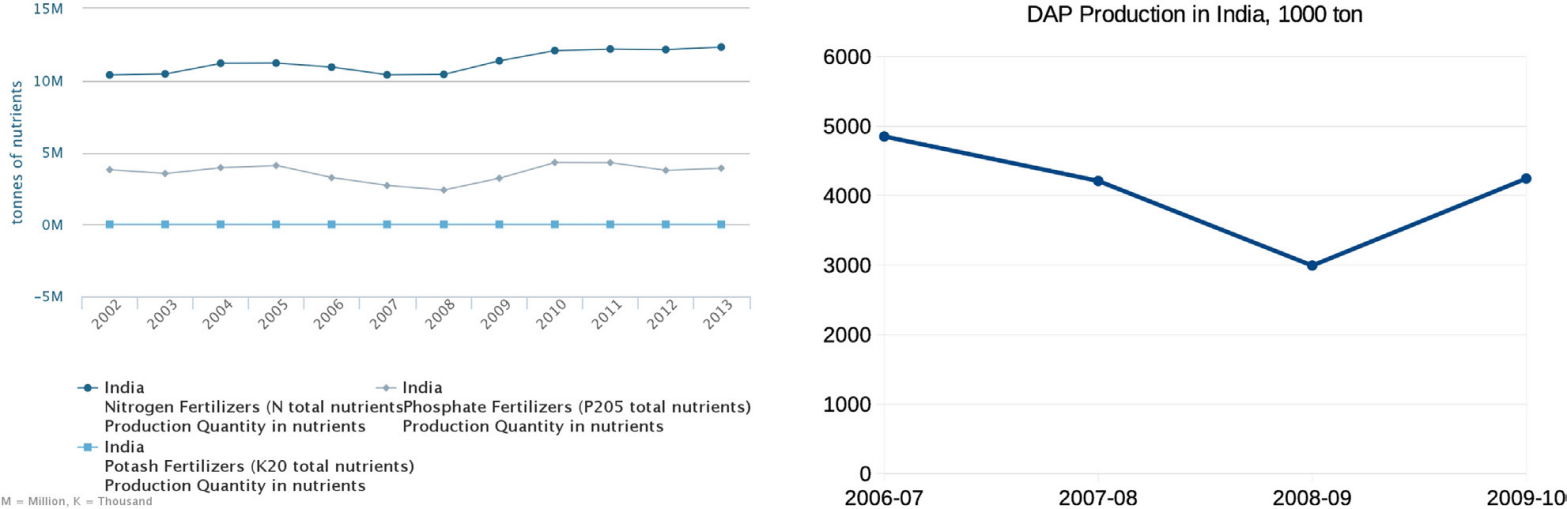
Production of fertilizers in India according to FAOSTAT (left panel) and specifically diammonium phosphate production in India according to the Department of Fertilizers of the Ministry of Chemicals and Fertilizers of India (right panel).

Indian agricultural producers in 2008 seem to have been in a very favorable situation. Below we analyze the Indian domestic fertilizer producers’ environment. According to the FAOSTAT data, throughout the entire 2005–2008 period, Indian fertilizer production was in decline with the largest decline (about 50% over that entire period) in phosphorus fertilizer (Figure 4). The data provided by the Department of Fertilizers of the Ministry of Chemicals and Fertilizers of India demonstrate an even sharper drop in domestic DAP production in 2008 as compared to 2007: the relative change to the previous year was about −13% in 2007 and −29% in 2008 (Figure 4).

**Figure 5:**
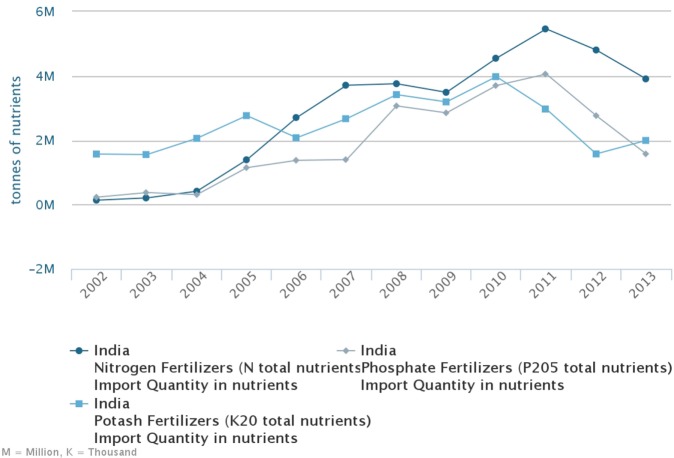
Indian import of fertilizers N, P, and K (total nutrients). Source: FAOSTAT.

An analysis of the import, production, and consumption can help better illuminate farmer behavior in the year of the price peak. Taking into account that the import of P-fertilizer was at a stable level over 2005–2007 as illustrated by Figure 5, the production gap in 2007–2008 (Figure 4) led to the accumulated deficit in domestic DAP production of 2.5 Mtons (using 2006 level as the baseline). The increase of DAP import over the period of 2007–2008 was 1.8 Mtons, estimated with the data from Comtrade (1). Hence, over 2007–2008, Indian farmers experienced a deficit in DAP of about 2.5 − 1.8 = 0.7 Mtons (unless it was compensated by reserves).

**Figure 6:**
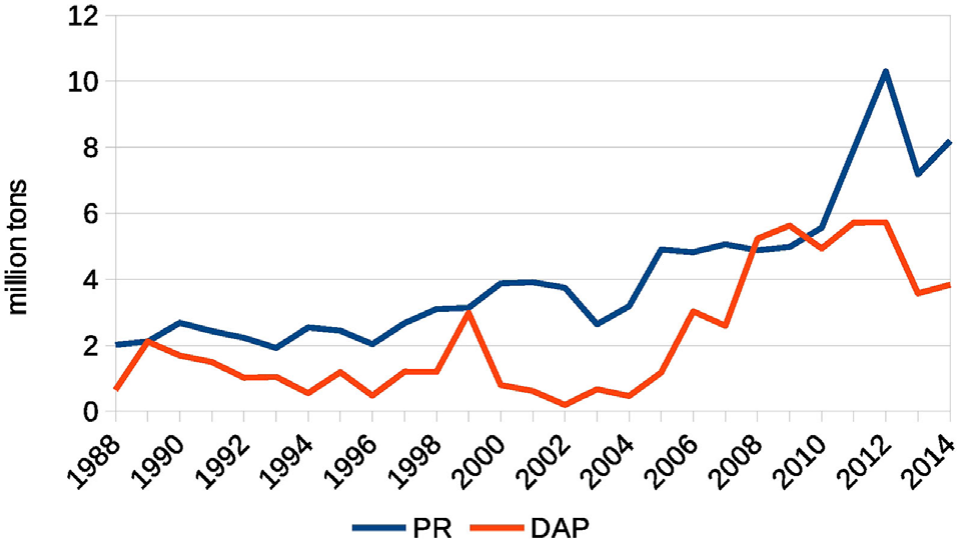
Indian import of phosphate rock (PR) and diammonium phosphate (DAP). Source: Comtrade.

Based on FAOSTAT data (8) on Indian import, this estimate is different: the total reported import increase in P_2_O_5_ equivalent over 2007–2008 (using 2006 as a baseline) was 1.7 Mtons. As the PR import stayed within this time frame at a constant level (see Figure 6), this increase can be attributed to a ready-made fertilizer (DAP, MAP, complex fertilizer) import increase, so that the amount of fertilizer in DAP equivalent would be approximately +3.4 Mtons employing IFA’s (7) conversion factor of about 50% content of P_2_O_5_ in DAP/MAP. As the Indian-imported MAP quantity is about 10% of that of DAP, the increase of DAP quantity imported over 2007–2008 using FAOSTAT leads to an estimate of 3.1 Mtons. This figure is able to compensate the reported by India 2.5 Mtons drop in DAP production and even indicates a 0.6-Mtons surplus in DAP. In fact, this number is likely smaller as we have accounted for complex fertilizers within this DAP equivalent. So, the estimated range is −0.7 … +0.6 Mtons of DAP for farmer use over the period 2007–2008. The up to +0.6 Mtons overall usage increase possibly caused by a higher demand from farmers is comparatively small as it is only 20% of the import increase (0.6/3.1 Mtons) as compared to the rest 80% caused by the deficit in domestic DAP production (2.5 Mtons) using 2006 level as a baseline. The farmers’ increase in P-fertilizer use in 2008 as reported by FAOSTAT (8) and illustrated in Figure 2 is even less—only about 10%.

The highlighted differences between FAOSTAT and Comtrade serve the purposes of estimating the farmers’ behavior. Employing Comtrade data that provides a lower estimate of Indian DAP import increase as compared to FAOSTAT does not lead to different conclusion on the role of India as compared to China. To support this statement, we use Comtrade data on DAP and in what follows we use the plus sign “+” to indicate increased availability on the market and minus sign “−” to indicate decreased availability on the market caused by a trade partner. First, referring to Table 1, the change in traded quantities are China +1.2 Mtons and India −1.8 Mtons accumulated over 2007–2008 using 2006 as a baseline,^2^ meaning that aggregated over 2 years, China was rather lowering the price by supplying additional quantity. Second, respective numbers in the year 2008 using 2007 as a baseline are China −1.2 Mtons and India −2.6 Mtons, meaning that the Indian impact in terms of quantity is more than two times bigger than that of China. Third, the protective measures China took in 2008 were a response to the already increasing fertilizer prices (10) rather than triggering them. The aggregates above are based on the original numbers that countries reported as exported (China) and imported (India) summarized in Table 1. A cross check for each DAP trade reported in Comtrade between any two countries and respective correction for possible omissions by taking the maximum of the two reported quantities (one for importer and one for exporter) would make the estimates even more pronounced: China +1.5 Mtons and India −2.2 Mtons over 2007–2008 (2006 as a baseline) and China −1.0 Mtons and India −3.1 Mtons in the year 2008 (2007 as a baseline). Taking into account the fact that over 2006–2008, China was also importing DAP, and overall reduced its import and increased export, Chinese numbers can be further adjusted and expressed in terms of net export impact: +3.7 Mtons over 2007–2008 (2006 as a baseline) and only −0.6 Mtons in the year 2008 (2007 as a baseline). These possible adjustments of the original numbers reported in Comtrade emphasize the major role of India in terms of demanded DAP quantity on the international market in 2008.

**Table 1:** Aggregated traded diammonium phosphate quantities for India and China over the period 2006–2008 as reported in the UN Comtrade database (1).

Year	China (export, Mtons)	India (import, Mtons)
2006	0.79	3.03
2007	1.97	2.59
2008	0.82	5.23

## Results

Out of wide set of factors quoted in the literature and quantitatively analyzed in this paper, the most decisive factor in the Indian DAP import increase as supported by the official statistical data was domestic production drop in 2007–2008. This led to an increase in demand in the world market by about 2.6–3.1 Mtons of DAP (affecting also MAP and complex fertilizers) in 2008 as compared to the previous year, while China increased DAP supply over 2007–2008 and took protective measures in 2008 reducing its export by 0.6–1.2 Mtons as compared to the previous year. Two consecutive drops in 2007 and 2008 in Indian domestic phosphate fertilizer production of, respectively, −0.5 and −2.0 Mtons of DAP (as compared to 2006 production level), which is about 5 and 20% of global DAP market, respectively, had likely triggered the P-fertilizer price spike of 2008 affecting other fertilizers, or at least had a decisive impact on the price spike magnitude. Protective measures of DAP exporters lead to a tighter supply and magnified the price spike. The potential response of Indian farmers to growing prices was buffered out by a price-agnostic subsidy scheme that also magnified the peak.

The reason for the Indian production drop was highly unfavorable conditions created for domestic producers as was later reported by the Department of Fertilizers: “The fertilizer sector worked in a highly regulated environment with cost of production and selling prices being determined by the Government of India. The growth of fertilizer industry was stagnated with virtually no investments … for over eight years in P&K sector” (18). The lack of a subsidy scheme supporting fertilizer produces symmetric to the one targeting fertilizer consumers resulted in the production drops in 2007–2008. So, the agricultural subsidies targeting farmers failed on another end—in the fertilizer production, as these supportive measures were isolated from the domestic fertilizer industry.

## Discussion

The failure in domestic policy resulted in a substantial impact outside of India in international fertilizer markets. A doubling of DAP demand within 1 year by the largest world importer, virtually without limits on the purchasing price, is very likely to have led to the observed market distortion. Moreover, as the fertilizer subsidy also covered product delivery, it is likely that the export price push was accompanied by a similar push to transportation prices, potentially affecting also the transportation market.

Interestingly, Indian farmers have not created an exceptionally strong additional demand being provided with a price guarantee and facing food price increase on the international market—they have increased consumption in 2008 by about only 10%, which is comparable to some previous and later years when the subsidy scheme changed.

Demand coming from the farmers’ side seems to be quite inelastic. The consequences of this inelasticity for the price dynamics in 2008 were amplified through national protective measures (China introducing export tariffs), tightening supply, and leading to higher prices. However, the respective impact is of secondary magnitude compared to the demand increase in the international market.

According to the Department of Fertilizers (19), “indigenous rock phosphate supplies meet only 5–10% of the total requirement of P_2_O_5_” in India, so the country is highly dependent on import of this raw material. According to Comtrade (1), India demonstrated a stable PR import at an approximately constant level through 2005–2008. At the same time, according to Comtrade (1), Morocco reported decrease its export in 2008 by −39% of DAP to the previous year (−4% of global supply) and −18% of PR (−9% of global supply). The situation is not completely clear as the respective counterparts report only −4% decrease of the import from Morocco. Since some importers might not have reported their trade for political reasons connected with the Western Sahara territory, and as PR production (mining) in Morocco was stable according to USGS, this probable drop in PR export may have supported the P price increase. In this context, a deeper analysis of the global PR supply dynamics might be worthwhile.

Another obvious fact to note is that the ultimate beneficiaries of the observed market distortion in 2008 were the fertilizer producers who collected outstanding profits in 2008. Taking into account high concentration of mining and production and common interest in profit maximization of a handful of big companies, one cannot exclude the possibility of their cooperation in supporting growing prices even without explicit coordination. Mosaic’s operating margin increased in 2007–2008 from 10% to almost 30% (13). According to Potash Corp. (20), their gross margin almost tripled in 2008 as compared to a quite successful year 2007. As fertilizers are important inputs in modern agriculture and ultimately impact food security, the consequences of a fertilizer market distortion might have potentially heavy impacts, especially when such a distortion duration lasts over several years. Therefore, there is a strong need for coordinated policies on national and international levels to provide stable conditions for all parties.

A note of caution on the conclusions obtained in this analytical work is that even though these are based on the data from the official and openly available sources such as FAOSTAT (8) and the UN Comtrade (1), these sources are known to be inaccurate and the data contained therein may be inconsistent as illustrated above for the Indian P-stock balance estimation. These conclusions would be subject to change if there were a major revision of the numbers provided by these sources.

## Author Contributions

MO provided the concept of the work. NK worked on data acquisition and drafted the manuscript. Both MO and NK carried out the analysis, interpretation of data, and revised the manuscript.

## Conflict of Interest Statement

The authors declare that the research was conducted in the absence of any commercial or financial relationships that could be construed as a potential conflict of interest.

## Footnotes

^1^http://www.fao.org/worldfoodsituation/foodpricesindex/en/.

^2^The calculation for China of a 2-year accumulated excess of supply using 2006 as a baseline is: 1.97 + 0.82 − 2 × 0.79 = 1.21 Mtons. The calculation for India is similar.
